# Prof. Severance Tells How He Keeps from Ever Getting Sick

**Published:** 1888-06

**Authors:** 


### PROF. SEVERANCE TELLS HOW HE KEEPS FROM EVER GETTING SICK.

Prof. A. B. Severance, the distinguished musician and dancing master, is a well preserved man of 64 years. He has a bright eye, elastic step, handsome face, long, flowing white beard, and hair of the same color that falls in heavy locks upon his shoulders. He is a man who will attract attention in any crowd. He is a fine conversationalist, and has some queer notions, but he can't be called a crank for he never obtrudes his opinions upon others unless he is asked to give his ideas about various social matters. A reporter of the *Review*, desiring to learn how he kept so healthy and well preserved, interviewed the gentleman on the subject.

"So you want to know how I live?" said the professor in answer to a question. Well, (we, that is, my wife, Dr. Juliet H. Severance and I) never eat meat of any kind, no fine wheat bread, no pickles or spices of any kind, no pie, cake or pastry, of any description, and we never drink tea, coffee, liquor or even water at meal time. What do we live on? Well, we eat two meals a day—one at 12 o'clock noon and the other at 6 o'clock in the evening and we never eat a particle between meals. We partake of Graham flour bread, baked in various ways, oatmeal, cracked wheat, rice, potatoes and all kinds of vegetables, cooked in a great many different ways, and we never fail to have fruit of some kind on the table. We are vegetarians and you can readily see that the plan agrees with us for we are never sick. I have lived that way for twenty-five years, and I have never been sick a day in all that time. I have never been forced to break an engagement on account of any physical ailment. I can walk a long distance without getting tired and I am in good trim throughout. I am a living example of the vegetarian theory. I do not use tobacco in any form and am strictly temperate in everything, but still I don't believe in prohibition, because I believe that every person should be allowed to do as he pleases in the matter of eating and drinking."

